# Hybrid and Total Endovascular Solutions for Aortic Arch Disease: Contemporary Surgical Strategies

**DOI:** 10.3390/jcm15051946

**Published:** 2026-03-04

**Authors:** Ermal Likaj, Saimir Kuci, Alfred Ibrahimi, Marsela Goga, Alvi Cela, Paolo Zamboni, Jacob Zeitani

**Affiliations:** 1 Cardiac Surgery Department, Mother Teresa University, 1000 Tirana, Albania; 2 Anesthesiology and Reanimation Department, Mother Teresa University, 1000 Tirana, Albania; 3 Vascular Diseases Center Unit, University of Ferrara, 44121 Ferrara, Italy; 4 Neurosciences and Rehabilitation Department, University of Ferrara, 44121 Ferrara, Italy

**Keywords:** aortic arch, hybrid repair, endovascular therapy, frozen elephant trunk

## Abstract

Aortic arch diseases represent one of the most complex domains in cardiovascular surgery due to the need for cerebral protection, anatomical precision, and durable reconstruction. Their management requires highly individualized strategies that balance cerebral protection, systemic organ perfusion, anatomical complexity, and long-term durability. Over recent decades, antegrade cerebral perfusion has significantly reduced neurological morbidity. In parallel, vascular prostheses have progressed from conventional Dacron grafts to hybrid stent graft systems simplifying arch reconstruction and expanded treatment options in high-risk cohorts. Total endovascular arch repair using branched or fenestrated devices has emerged for selected high-risk patients; however, current data remain limited, with early stroke rates of approximately 5–8% and restricted long-term durability evidence. From both clinical and economic perspectives, open and hybrid approaches remain central to durable arch management.

## 1. Introduction

Aortic arch disease, including aneurysms and dissections, remains among the most technically demanding conditions in cardiovascular surgery due to the arch’s complex geometry, the need for meticulous cerebral protection, and the high risk of neurological and systemic complications [1]. Unlike pathology confined to the ascending or descending aorta, arch disease mandates simultaneous control of cardiac, cerebral, and systemic circulation, making strategic planning as critical as technical execution. Although isolated arch aneurysms are relatively uncommon, most patients present with multisegment disease extending into the ascending and/or descending aorta, requiring integrated rather than segmental treatment strategies. Over the past five decades, arch surgery has evolved profoundly through advances in extracorporeal circulation, cerebral protection, and graft technology. The introduction of deep hypothermic circulatory arrest (DHCA) and later antegrade cerebral perfusion (ACP) significantly improved neurological outcomes and procedural safety [2,3,4,5,6]. These refinements, combined with improvements in perfusion strategies and intraoperative monitoring, have reduced perioperative morbidity and resource utilization, reinforcing the link between technical excellence and economic sustainability in modern arch repair [7,8,9,10,11,12]. A major conceptual shift occurred with the introduction of the elephant trunk (ET) technique and its subsequent evolution into the frozen elephant trunk (FET). By integrating open arch replacement with antegrade stent graft deployment into the descending thoracic aorta, the FET enables single-stage treatment of extensive disease while creating a durable proximal landing zone for future endovascular interventions [13,14,15,16,17,18]. The FET thus serves both as definitive surgical reconstruction and as an enabling strategy for staged endovascular therapy. In parallel, hybrid arch repair combining supra-aortic debranching with TEVAR was developed to reduce physiological stress in selected patients [19,20]. More recently, total endovascular arch repair using branched or fenestrated devices has emerged as a minimally invasive option in high-risk cohorts [1]. Notably, unlike the descending thoracic aorta, the aortic arch lacks long, cylindrical landing zones and is characterized by continuous motion, asymmetric curvature, and critical neurovascular outflow, factors that fundamentally limit the generalizability of endovascular paradigms developed for infrarenal or descending thoracic pathology. However, unlike the descending thoracic aorta, the arch remains a domain where anatomy, neuroprotection, and durability impose fundamental limits on purely endovascular solutions. High device costs, complex arch geometry, cerebrovascular risk, and reintervention burden constrain widespread application. Collectively, these developments do not represent a linear transition from open to endovascular therapy, but rather an evolution toward integrated, anatomy-driven strategy selection. In this paradigm, surgery is not replaced by endovascular therapy; rather, it enables it. Open and hybrid approaches remain indispensable, providing anatomical control, procedural safety, and long-term durability while facilitating safe endovascular innovation.

## 2. Elephant Trunk and Frozen Elephant Trunk (FET)

By avoiding a single extensive thoracic procedure, the staged ET approach significantly reduced the operative burden associated with prolonged cardiopulmonary bypass, deep hypothermia, and extended circulatory arrest, thereby improving early survival in patients with complex aortic disease [13,14,15]. However, from a strategic standpoint, the conventional ET should be viewed as a temporizing solution rather than a definitive one, given the well-documented attrition between stages. To overcome these limitations, the frozen elephant trunk (FET) technique was developed in the 1990s. The FET combines open aortic arch replacement with the simultaneous antegrade deployment of a stent graft into the descending thoracic aorta, allowing true single-stage treatment of combined arch and descending aortic pathology. This innovation effectively collapses a two-stage philosophy into a single operative strategy, reducing cumulative risk while enhancing anatomical control. Meta-analyses and large contemporary registries have consistently demonstrated that the FET provides superior false lumen thrombosis and reduced downstream reintervention compared with the conventional ET in both aneurysmal and dissecting pathology, albeit at the cost of increased procedural complexity requiring experienced centers. By delivering the stent graft under direct visualization through the open arch, the FET eliminates the need for a second thoracotomy and significantly reduces interval mortality compared with the conventional ET procedure [15,17]. Beyond its technical advantages, the FET has reshaped long-term aortic management by converting complex multisegment disease into a modular platform for staged endovascular extension when required. This “surgery as infrastructure” concept is increasingly recognized as central to durable arch therapy.

### 2.1. Evolution of the Frozen Elephant Trunk: From Straight Stent Grafts to Branched and Next-Generation Devices

**Figure 1:**
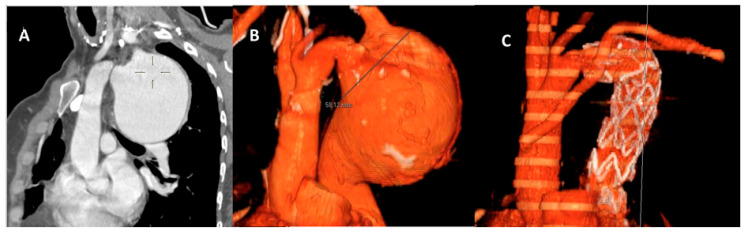
Pre- (**A**,**B**) and postoperative (**C**) CT Scan images showing large thoracic aneurysm involving the distal arch and descending aorta, where a surgical hybrid prosthesis is deployed in the descending aorta. Distal anastomosis is performed in zone 2 and extra-anatomic left subclavian artery bypass.

**Figure 2:**
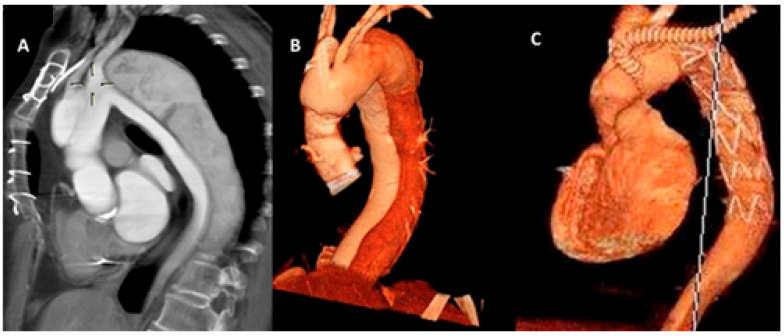
Preoperative (**A**,**B**) and postoperative (**C**) CT scan images showing chronic dissection involving the aortic arch and descending aorta with prominent false lumen. The distal hybrid graft anastomosis was performed in zone 2, with implantation of two supra-aortic vessels using the island technique and an extra-anatomic left subclavian artery (LSA) bypass.

**Figure 3:**
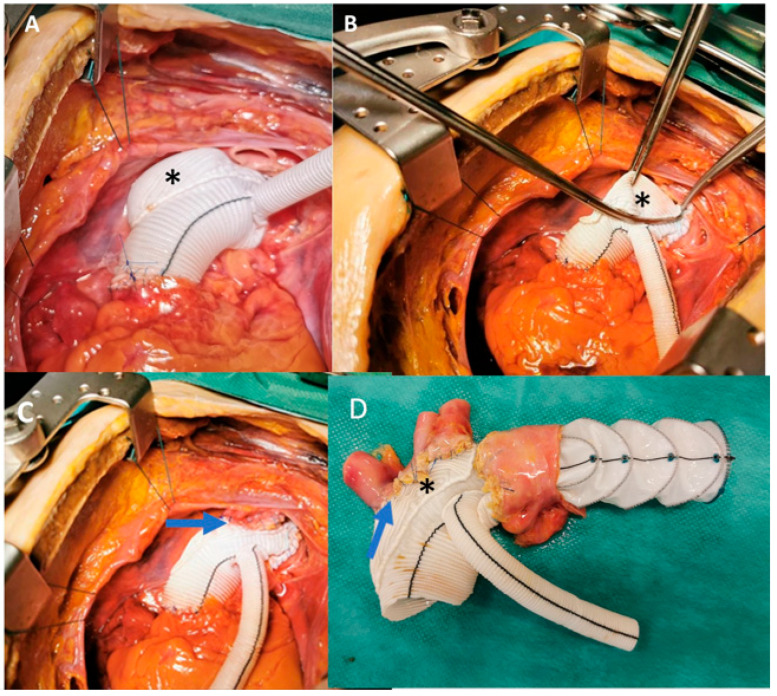
In a cadaveric model, the Thoraflex™ island hybrid graft is anastomosed to the distal aortic arch and ascending aorta (**A**). A partial clamp is applied to the extended Dacron cuff (*) to enable en bloc anastomosis of the supra-aortic (epiaortic) vessels) (**B**). Final configuration of the island technique for supra-aortic vessel implantation (arrow) using the Thoraflex™ island hybrid graft (**C**). Explanted prosthesis showing the supra-aortic vessels (arrow) and distal anastomoses sites (**D**).

The frozen elephant trunk (FET) technique has undergone substantial technological evolution since its inception, reflecting both expanding clinical ambition and increasing anatomical complexity [18,19]. Early FET systems, such as the E-vita Open and Thoraflex Hybrid, were based on straight, non-branched stent graft designs integrated with a proximal surgical Dacron graft. These first-generation devices were primarily intended to facilitate single-stage treatment of combined arch and descending thoracic aortic disease, simplify distal anastomosis, and promote false lumen thrombosis in dissection. While they established the fundamental value of FET as a hybrid surgical–endovascular solution, they remained dependent on conventional arch reconstruction with separate and technically demanding reimplantation of the supra-aortic vessels [20]. As experience accumulated and indications expanded, branched FET designs were developed to streamline arch reconstruction, reduce cerebral and myocardial ischemic times, and improve procedural reproducibility. The introduction of multibranched surgical collars allowed en bloc or separate reattachment of the supra-aortic vessels, improving anatomical alignment, and facilitating controlled cerebral perfusion strategies. More recently, next-generation devices such as the neo E-vita open have been specifically designed to facilitate proximalization of the distal anastomosis and simplify supra-aortic vessel reconstruction, addressing two of the most challenging technical aspects in complex arch surgery [21]. Proximalization, moving the distal suturing site closer to the ascending aorta or arch zones 1–2 (Figure 1), represents a major technical and strategic advancement, reducing operative time, limiting circulatory arrest, and simplifying reconstruction in the most hostile portion of the arch [22,23,24]. This approach transforms the distal arch from an exposure-limited, high-risk zone into a controlled and reproducible operative field, particularly valuable in elderly patients, redo procedures, and acute dissections (Figure 2). By integrating a multibranched surgical collar with an optimized stent graft component, the hybrid graft enables controlled proximal arch replacement, reproducible head vessel reimplantation, and reduced operative complexity, cerebral ischemic times, and procedural variability. However, both the traditional en bloc island technique and the branched graft technique (BGT) have recognized advantages but also important limitations in aortic arch surgery [25,26,27]. To combine the benefits of both while overcoming their respective drawbacks, a novel island graft has been developed. This prosthesis consists of a hybrid graft with an additional superior “bubble” for en bloc island anastomosis, allowing distal anastomosis in zone 2 or 3 while preserving native branch geometry [28]. The island graft was conceived to facilitate implantation with reduced manipulation of epiaortic vessels, potentially lowering neurological risk, while allowing tailored fenestration of the external cuff to the island segment, early body reperfusion after distal anastomosis, and elimination of most pathological aortic tissue (Figure 3). Early preclinical experience with island-type hybrid grafts suggests potential reductions in supra-aortic vessel manipulation and cerebral ischemic time.

### 2.2. FET Complications

In chronic aortic dissection, longer stent graft segments are often preferred to achieve extensive coverage of the diseased aorta, enhance distal sealing, promote favorable aortic remodeling, and minimize the risk of distal stent-induced new entry (dSINE) [29]. The rationale is that a rigid, adequately sized stent provides radial support to the chronically remodeled aortic wall, facilitating thrombosis of the false lumen and stabilizing distal segments. In acute aortic dissection, however, therapeutic priorities are fundamentally different. The objective is not maximal distal coverage but strategic proximal stabilization. Treatment aims to control the primary entry tear, restore true lumen perfusion, and establish a durable proximal landing zone, while carefully limiting distal extension to avoid complications. Excessive distal coverage in the acute setting can increase the risk of spinal cord ischemia (SCI), especially in patients with compromised collateral circulation, and may predispose to dSINE due to the fragile, acutely dissected distal aortic wall. This distinction highlights the importance of a pathology-driven FET strategy: device selection and stent graft length should be tailored to the dissection chronicity, aortic anatomy, and target objectives rather than applying a uniform distal coverage approach. In acute dissection, prioritizing controlled proximal repair with careful consideration of stent diameter, radial force, and distal length is critical to balancing effective aortic stabilization with preservation of spinal cord and distal aortic integrity [30,31]. Spinal cord ischemia (SCI) remains one of the most feared complications during extensive arch and descending thoracic aortic interventions, including both open surgical FET procedures and hybrid TEVAR extensions. Risk is multifactorial and increases with long-segment aortic coverage, prior aortic surgery, occlusion of intercostal or hypogastric arteries, and compromised collateral networks. Prophylactic cerebrospinal fluid (CSF) drainage combined with continuous spinal pressure monitoring enables optimization of spinal cord perfusion pressure (mean arterial pressure minus CSF pressure) intraoperatively and in the early postoperative period. Immediate correction of blood hypotension and prompt drainage in the presence of elevated CSF pressure are critical to prevent irreversible ischemic injury [32,33]. When incorporated into standardized perioperative protocols, including staged aortic coverage, permissive hypertension, and early neurological surveillance, SCI rates have been reduced from historical levels of 10–20% to below 5% in contemporary series [34,35]. Nevertheless, the risk of delayed spinal cord ischemia persists, particularly following secondary distal endovascular extensions, underscoring the importance of staged strategies, collateral preservation, and extended postoperative neurological vigilance. These data reinforce that spinal protection in the FET is not an adjunct but a central strategic determinant of procedural design and execution.

## 3. From Straight Frozen Elephant Trunk to Branched and Total Endovascular Arch Repair: Escalating Complexity at Neurovascular Crossroads

The management of complex aortic arch disease has evolved substantially over the past two decades, driven by advances in device technology and a growing appreciation of the unique anatomical, hemodynamic, and neurological constraints of the arch. The FET technique marked a pivotal step in this evolution. Rather than representing a transition away from surgery, the FET established a durable surgical–endovascular interface that redefined strategic planning for extensive arch disease [36,37,38,39]. However, unlike the descending thoracic aorta, the arch represents a neurovascular crossroads in which uninterrupted cerebral perfusion, precise anatomical reconstruction, and durable hemodynamic performance are mandatory rather than adjunctive considerations. The arch is characterized by pronounced curvature, short and angulated landing zones, and continuous pulsatile and torsional motion. These factors increase susceptibility to bird-beaking, Type I endoleak, migration, and incomplete sealing, failure modes that, in the arch, may carry catastrophic neurological consequences [40,41]. Specifically, Type IA endoleak is the most commonly reported complication in branched and fenestrated arch devices, occurring in 3–10% of cases, where incomplete apposition of the proximal stent graft creates a channel for blood flow to re-enter the aneurysm sac. Stent graft migration has been observed in 2–5% of patients, with downstream displacement potentially resulting in late aneurysm expansion or rupture. Device collapse or infolding, typically caused by excessive radial force along the lesser curvature of the arch, has been reported in up to 1–3% of cases, leading to compromised flow or end-organ perfusion. Aneurysm expansion or rupture secondary to persistent endoleak remains a serious albeit infrequent complication (<2%), and material fatigue or stent fracture has been documented in 1–2% of long-term follow-up studies, reflecting structural stress imposed by the complex arch geometry [41,42]. Therefore, total endovascular repair of aortic arch pathology using multibranched stent graft systems remains among the most technically demanding interventions. Beyond geometry, the arch is a region of complex three-dimensional blood flow characterized by high shear stress, secondary vortices, and marked pulsatility. The introduction of multibranched stent grafts fundamentally alters native flow patterns and may generate regions of flow separation, turbulence, and low wall shear stress, conditions known to favor thrombus formation. Computational fluid dynamics and in vivo imaging studies have demonstrated that even subtle deviations in branch angulation or diameter mismatch can generate zones of low wall shear stress and flow recirculation, which are mechanistically linked to thrombus formation and embolic risk in branched arch devices. These disturbances are particularly relevant at branch takeoffs, within curved branch grafts, and at branch–main body junctions, where geometric mismatch, angulation, or length discrepancies can impair laminar flow and promote blood stagnation. From an anatomical and biomechanical standpoint, the aortic arch is a highly dynamic structure subjected to continuous pulsatile deformation and multidirectional forces [42,43]. Endovascular devices must achieve intimate wall apposition while maintaining conformability across variable diameters and curvatures. Excessive rigidity may lead to kinking or incomplete sealing, whereas insufficient radial force risks migration or loss of fixation. These issues are magnified in the proximal arch and ascending aorta, where landing zones are short and angulated, and excessive radial force may precipitate retrograde dissection or device-induced injury [44]. Precise alignment of multiple branches with the supra-aortic vessels is a defining challenge of total endovascular arch repair. Three-branched devices must accommodate the brachiocephalic trunk, left common carotid artery, and left subclavian artery, each exhibiting patient-specific variability in origin, angulation, and diameter. Even minimal deployment inaccuracies can result in impaired cerebral perfusion or technical failure, and unlike with fenestrated abdominal devices, opportunities for post-deployment correction are extremely limited [45]. Branch graft kinking, compression, or excessive curvature represents a major failure mode in total endovascular arch repair. Even subtle deviations in branch orientation may initially remain clinically silent yet predispose individuals to delayed thrombosis or thromboembolic events. In contrast to open or hybrid reconstructions, endovascular branch patency relies entirely on sustained physiologic flow conditions, making hemodynamic optimization a critical determinant of long-term success. Thromboembolic risk therefore represents a central concern in total endovascular arch repair. Thrombus formation within branch grafts, at junctional zones, or along the inner curvature of the main stent graft may result in cerebral or upper-extremity embolization, with potentially devastating neurological consequences. This risk is amplified by prolonged procedural times, extensive prosthetic surface area, repeated branch cannulations, and the need for complex anticoagulation and antiplatelet regimens that must balance competing risks of thrombosis and bleeding.

The NEXUS Aortic Arch Stent Graft System represents a purpose-built, arch-specific endovascular designed for zone 0 deployment. that has evolved from an initial single-branch configuration requiring surgical debranching of the left-sided supra-aortic vessels to the dual-branch Nexus Duo and, more recently, to the customizable NEXUS TR aimed at fully endovascular arch reconstruction. Unlike off-the-shelf thoracic devices adapted for proximal use, NEXUS consists of a two-component modular system with a proximal arch module and a distal descending thoracic module connected via a docking sleeve, allowing independent alignment and conformability within the curved arch. Based on company-reported data from an investigational device exemption (IDE) study, the prospective, multicenter TRIOMPHE investigational device exemption study evaluated the single-branch NEXUS configuration, enrolling 94 high surgical-risk patients with zone 0 arch pathology [46]. While lesion-related mortality beyond 30 days was 0% through 1 year, early adverse events were observed, including a 30-day procedural related mortality of 6.4% and a disabling stroke rate of 7.4%, predominantly occurring after device deployment. However, endoleak complications were reported to be low, with only three cases of Type 1a or Type 1b, and no Type III or IV endoleaks were detected. Importantly, the TRIOMPHE study evaluated the single-branch NEXUS configuration, incorporating only the brachiocephalic trunk, with surgical management of the remaining supra-aortic vessels. Extrapolation of these outcomes to dual-branch or total arch configurations should therefore be approached cautiously. Speculatively, extension of endovascular manipulation to both carotid arteries not only may increase procedural cerebral embolic risk due to longer arch dwell time and repeated cannulation, but may also alter main graft conformability, fixation, and rotational stability within the curved arch, with potential secondary effects on sealing, branch alignment, and native arch flow patterns, reinforcing the need for rigorous neurological surveillance and prospective evaluation. In parallel with the evolution of the NEXUS device, other manufacturers have pursued endovascular arch solutions. The Relay Branch Thoracic Stent-Graft System (Terumo Aortic, Glasgow, United Kingdom) has been developed as an arch-specific branched endoprosthesis, available in single-, double-, and triple-branched configurations, designed for total endovascular reconstruction of the aortic arch with modular branch alignment and proximal-to-distal deployment flexibility. Early outcome reports for RELAY™ devices demonstrate technical success rates exceeding 90% with 30-day procedural related mortality in the range of approximately 6–9% and disabling stroke rates of approximately 5–8%, comparable to early experience with other branched systems in high-risk cohorts. [47] To provide a balanced comparison across strategies, contemporary data from high-volume centers indicate that elective open total arch replacement is associated with a 30-day mortality ranging from approximately 4 to 8%, with disabling stroke rates between 5 and 10%. Frozen elephant trunk (FET) procedures report early mortality rates of 5–10% and permanent neurological deficit in 4–8% of patients, depending on urgency and extent of repair. Hybrid arch repair demonstrates comparable early outcomes, with 30-day mortality typically reported between 6 and 12% and disabling stroke rates of 6–11%, reflecting the combined burden of surgical debranching and endovascular manipulation. These figures underscore that, although total endovascular approaches aim to reduce surgical invasiveness, early mortality and neurological complication rates remain within a similar range across open, hybrid, and branched endovascular strategies, particularly in high-risk or redo cohorts. Therefore, procedural selection should be guided primarily by anatomical suitability, institutional expertise, and long-term durability considerations rather than by perceived differences in early event rates alone [4,17,19,20]. Cook Medical’s Zenith family includes established thoracic and abdominal endovascular grafts such as the Zenith Alpha thoracic and fenestrated Zenith devices for aneurysm and dissection indications, though these are not specific for total arch reconstruction [48]. Historically, Cook developed a customized arch branch graft concept designed to seal in the ascending aorta with internal branches to the supra-aortic vessels. The Zenith arch branch concept integrates inner branches within a single main-body graft, requiring complex catheterization from upper extremity access. this investigational design remains outside of routine commercial use and is not widely supported by prospective clinical outcomes [49]. The relative absence of large, prospective, multicenter outcome data for arch-specific Zenith branch concepts contrasts with the more structured IDE experience reported for other platforms, limiting direct comparative assessment and reinforcing the investigational nature of total endovascular arch reconstruction across manufacturers. In contrast, the GORE^®^ TAG Thoracic Branch Endoprosthesis (TBE) was initially developed for zone 2 pathology, preserving left subclavian perfusion through a single retrograde branch. Its design differs fundamentally from NEXUS in that it incorporates a side-branch portal within a standard thoracic stent graft rather than a modular arch-docking system. Proximal extension into zones 1 and 0 expands applicability but requires mandatory surgical debranching, externalizing complexity to the open stage [50]. While TBE demonstrates excellent distal conformability and sealing, its use in proximal zones does not obviate hybrid or open intervention, underscoring the trade-off between device integration and surgical burden. However, adequate proximal landing zone is crucial to reduce the risk of Type I endoleak. Prior ascending aorta replacement may provide a prosthetic landing zone, yet graft kinking, angulation, or diameter mismatch can render even a Dacron graft geometrically hostile, emphasizing that surgical preparation is decisive [51]. Recent multicenter registry data reinforce this principle. In the Italian Nexus Aortic Arch Endovascular Repair Registry (INARCHER), 31 patients (mean age of 73.4 years; 48.4% with prior ascending surgery) achieved 97% technical success and 100% freedom from aortic-related mortality at 30 months, with one Type I endoleak requiring early reintervention [49]. While these results confirm the feasibility of total endovascular arch repair in selected patients, low procedural volumes, neurological risk, and persistent seal concerns highlight the continued reliance on careful surgical preparation [52]. In this context, redo intervention after previous ascending aorta replacement represents a distinct clinical scenario in which prior surgical reconstruction may paradoxically simplify subsequent arch or proximal descending repair. Proximalization of the distal anastomosis and surgical debranching of the epiaortic vessels can significantly streamline the redo procedure, creating a stable proximal landing zone and allowing treatment with a standard thoracic endovascular aortic repair (TEVAR) rather than a more complex hybrid arch graft. This strategy may reduce operative complexity, cardiopulmonary bypass time, and overall procedural costs by avoiding custom or branched hybrid devices. Indeed, leaving a few centimeters of the previous Dacron graft at the distal anastomosis offers several technical advantages when employing either TEVAR or hybrid stent grafts. First, the anastomosis between two prosthetic grafts is technically easier because the diameters of the old and new Dacron grafts are usually comparable. In contrast to the native aortic wall, the structural resistance and uniformity of Dacron fabric allow for faster and more secure suturing. Moreover, the prosthetic landing zone provides predictable geometry and improved sealing characteristics compared with fragile or dissected native tissue. In cases of chronic dissection with prior ascending replacement, TEVAR is often preferred over hybrid arch grafting. The availability of different stent graft diameters and lengths, including tapered configurations, facilitates adaptation to the true lumen, potentially reducing oversizing and lowering the risk of stent-induced new entry tears. In this context, the previously implanted Dacron graft functions as a durable and stable proximal landing zone, enhancing procedural safety while simplifying the overall strategy, while simultaneously offering a more cost-effective alternative to hybrid arch devices [53].

## 4. Economic Considerations

Despite avoiding sternotomy and cardiopulmonary bypass, total endovascular arch repair entails substantial cumulative costs. Branched/fenestrated endografts are among the most expensive cardiovascular implants, requiring bridging stents, cerebral protection devices, hybrid OR infrastructure, prolonged fluoroscopy, and specialized teams. Complications, including access site events, stroke, and reinterventions, may negate potential reductions in length of stay. Arnes et al. reported a mean total technical cost of $105,164 ± $59,338, with reimbursement frequently insufficient, yielding negative net technical margins [54]. These findings emphasize that cost-effectiveness analyses limited to index hospitalization substantially underestimate the true economic impact of total endovascular arch repair, particularly when lifelong imaging surveillance, secondary interventions, and stroke-related disability are incorporated [55].

Comparative economic analyses suggest that while total endovascular arch repair may exceed $100,000 in device-related technical costs alone, reported index hospitalization costs for open total arch replacement typically range between $45,000 and $75,000 depending on length of hospital stay and related complications. FET-based procedures incur additional prosthesis-related costs compared with conventional open arch repair but remain substantially lower than cumulative branched endograft expenditures. Moreover, open and FET strategies have demonstrated lower mid-term reintervention rates in observational series, contributing to more predictable lifetime economic profiles when surveillance imaging, secondary procedures, and device-related complications are incorporated into long-term cost modeling.

By contrast, open repair and FET-based strategies, though resource-intensive upfront, offer predictable long-term durability, reduced reintervention, and stable lifetime costs. Hybrid repair occupies an intermediate position, reducing operative trauma while preserving anatomical control and enabling future endovascular extensions. From a lifetime cost perspective, durability is both a clinical and economic imperative, minimizing reintervention, neurological complications, and hospital readmissions. Strategic therapy selection must therefore integrate clinical, anatomical, and economic considerations, reinforcing that surgery, open or hybrid, remains the platform that maximizes long-term outcomes while aligning with sustainable healthcare resource stewardship [56].

## 5. Decision-Making for Aortic Arch Repair

Optimal management of complex aortic arch disease requires a structured, patient-centered approach that integrates anatomical complexity, physiological reserve, and procedural strategy, while considering resource utilization [57,58]. Open, hybrid, and total endovascular repair are not competing alternatives but complementary components of an anatomy-driven framework, with the overarching goal of creating durable aortic architecture that enables safe, reproducible interventions [59,60]. Open surgical repair remains the benchmark for geometric precision, structural stability, and long-term freedom from reintervention. It is particularly indicated in younger patients, those with connective tissue disorders, or with extensive multisegment disease. By restoring normal arch geometry and excising diseased tissue, open repair establishes a robust proximal foundation for downstream interventions. Beyond clinical outcomes, open repair provides predictable long-term cost profiles by minimizing reinterventions and prolonged hospitalization [61].

Hybrid repair occupies a strategic middle ground. Combining supra-aortic debranching with endovascular exclusion, it reduces physiological stress while maintaining control over arch geometry and creating reliable proximal landing zones for future interventions. Hybrid approaches are particularly valuable in redo cases, hostile anatomy, or patients at high surgical risk and function as an enabling procedure rather than a compromise, balancing procedural safety, technical efficiency, and long-term aortic remodeling [55,57,62,63].

Total endovascular repair using branched or fenestrated devices represents the most advanced transcatheter option, reserved for anatomically suitable patients with high surgical risk. Purpose-built devices, such as the NEXUS system, address arch-specific geometric and neurovascular challenges while internalizing procedural complexity. Success remains highly anatomy-dependent, and complications such as Type I endoleak, stroke, and retrograde dissection persist even in expert hands. Accordingly, patient selection should prioritize arch geometry, branch orientation, and cerebral collateral robustness over chronological age or procedural invasiveness alone. Critically, total endovascular repair is rarely standalone; it is most successful when enabled by prior or concurrent open or hybrid procedures that establish controlled proximal geometry and durable landing zones.

From a resource perspective, integrating open, hybrid, and endovascular approaches optimizes long-term outcomes, minimizes reintervention, and reduces cumulative healthcare costs, reinforcing the principle that surgery does not compete with endovascular therapy, it enables it.

## 6. Limitations and Conclusions

This review has several inherent limitations. Most evidence on hybrid and total endovascular arch repair derives from single-center series, feasibility studies, and registries, with limited randomized or long-term comparative data. Reporting standards are heterogeneous, and device durability beyond five to ten years remains incompletely defined. Furthermore, many contemporary total endovascular arch devices remain investigational or approved only in limited jurisdictions, restricting widespread adoption and emphasizing the need for long-term post-market surveillance. Rapid technological evolution, operator learning curves, and institutional volume further limit generalizability. Economic analyses are sparse and highly context-dependent, while patient selection bias, favoring endovascular approaches in high-risk cohorts, complicates direct comparisons.

Despite these limitations, the data reinforce a central principle: strategy should be anatomy-driven rather than device-driven. Open, hybrid, and total endovascular repair are complementary, not competing, modalities. Total endovascular repair provides a minimally invasive solution for high-risk patients but remains constrained by complex anatomy, neurological risks, low procedural volumes, and durability concerns. Open and FET-based strategies continue to deliver predictable long-term outcomes, reduced reintervention rates, and durable aortic reconstruction, whereas endovascular-only approaches carry higher device costs, increased resource utilization, and a greater likelihood of secondary interventions. Looking forward, technological advancements may gradually expand the role of total endovascular repair. Custom-made devices derived from preoperative computed tomography imaging could replicate native branch anatomy, improving branch alignment, reducing bird-beak configurations, and minimizing neurological and thromboembolic complications. Innovations in graft materials, lower-profile systems, and enhanced imaging guidance may further improve conformability, procedural safety, and applicability. These developments could allow select high-risk patients to undergo entirely endovascular repair with outcomes approaching those of open or hybrid procedures. Nevertheless, open and hybrid strategies will likely remain essential for complex anatomy, emergent cases, and situations requiring maximal durability.

In conclusion, contemporary management of the aortic arch requires strategically integrated, patient-centered decision-making, where optimal outcomes rely on stable proximal architecture, carefully staged hybrid or open procedures, and vigilant perioperative planning rather than on device sophistication alone. The evolution from straight FET to branched hybrid systems, and ultimately to total endovascular arch repair, reflects the ongoing effort to address the inherent anatomical complexity and high neurological stakes of this region. While total endovascular approaches offer promising alternatives for high-risk patients, their widespread adoption is limited by anatomical, technical, and device-related challenges. Continued innovation—focusing on improved conformability, reduced device profiles, precise branch alignment, and advanced imaging integration, will be essential to enhance the safety, durability, and applicability of these therapies. Ultimately, durable outcomes in aortic arch interventions depend more on architectural control and strategic planning than on the sophistication of the devices themselves.

## Data Availability

No new data were created or analyzed in this study. Data sharing is not applicable.
